# SUBJECTIVE SLEEP NEED AND DAYTIME SLEEPINESS IN ADOLESCENTS

**DOI:** 10.1590/1984-0462/;2019;37;2;00014

**Published:** 2019-02-25

**Authors:** Geraldo Jose Ferrari, Diego Grasel Barbosa, Rubian Diego Andrade, Andreia Pelegrini, Thais Silva Beltrame, Érico Pereira Gomes Felden

**Affiliations:** a Universidade do Estado de Santa Catarina, Florianópolis, SC, Brazil.

**Keywords:** Sleep, Sleepiness, Sleep deprivation, Adolescents, Sono, Sonolência, Privação de sono, Adolescente

## Abstract

**Objective:**

To analyze the contribution of subjective sleep need for daytime sleepiness in adolescents, and to compare questions about sleep, age and body mass index between adolescents who considered to sleep enough and those who reported the need for more sleep.

**Methods:**

This is a descriptive, epidemiological and cross-sectional study. Data collection was performed in August 2016, with 773 adolescents aged 14-19 years old, from Paranaguá, Paraná, Southern Brazil. The analysis included the following variables: time in bed, half-sleep phase, sleep need, social jetlag, daytime sleepiness, body mass index and physical activity.

**Results:**

The prevalence of adolescents with subjective need for sleep was 73.0%, with an average need of 1.7 extra hours of sleep. These adolescents woke up earlier (p<0.001) and slept less on school days (p<0.001). The need for more sleep was associated with higher daytime sleepiness scores (rho=0.480; p<0.001) and with later half-sleep phase (rho=0.200; p<0.001). No correlation was identified between the sleep need and time in bed (rho=-0.044; p=0.225). The subjective sleep need was the variable with the greatest explanatory power for daytime sleepiness (24.8%; p<0.001). In addition, the less adolescents practiced physical activity, the higher their daytime sleepiness scores (rho=-0.117; p<0.001).

**Conclusions:**

The subjective sleep need has an important role in explaining daytime sleepiness among adolescents. Adolescents who needed to sleep more reported waking up early and experienced sleep deprivation during class days; they also woke up later on the weekends and experienced more daytime sleepiness, compared to those who believed they had enough sleep.

## RESUMO

**Objetivo:**

Analisar a contribuição da necessidade subjetiva de sono para a sonolência diurna em adolescentes e comparar questões sobre sono, idade e índice de massa corporal entre os adolescentes que consideram dormir o suficiente e os que julgam necessitar dormir mais.

**Métodos:**

Estudo descritivo, epidemiológico e transversal, cuja coleta de dados ocorreu em agosto de 2016 em 773 adolescentes de 14 a 19 anos de Paranaguá, PR, Brasil. As variáveis de análise foram tempo de cama, meia fase do sono e necessidade de mais horas de sono, além do *jetlag* social, sonolência diurna, índice de massa corpórea (IMC) e atividade física.

**Resultados:**

A prevalência de adolescentes com necessidade subjetiva de sono foi 73,0%, sugerindo precisar, em média, de 1,7 hora a mais de sono. Estes acordavam mais cedo (p<0,001) e dormiam menos nos dias de aula (p<0,001). A necessidade de dormir mais associou-se a maiores pontuações de sonolência diurna (rho=0,480; p<0,001) e com meia fase do sono mais tardia (rho=0,200; p<0,001). Não foi identificada correlação entre necessidade de sono e tempo de cama (rho=-0,044; p=0,225). A necessidade subjetiva de sono foi a variável com maior poder explicativo da sonolência diurna (24,8%; p<0,001). Além disso, quanto menos os adolescentes praticavam atividade física, maiores as pontuações de sonolência diurna (rho=-0,117; p<0,001).

**Conclusões:**

A percepção subjetiva de sono teve papel importante para explicar a sonolência diurna dos adolescentes. Os jovens que precisavam dormir mais relataram acordar mais cedo e apresentavam privação de sono durante os dias de aula, acordaram mais tarde no final de semana e apresentaram maior sonolência diurna, comparando-se aos que acreditavam dormir o suficiente.

## INTRODUCTION

Adolescence has long been recognized as a period characterized by the progressive delay in sleep and wake-up times,^1^ and this delay is usually attributed to reduced control of time from the parents, increasing school chores, extracurricular activities, work, social events and use of computer and television in the evening.^2^ Besides, there is evidence that developmental changes in circadian rhythm delay the onset of sleep in puberty.^2^ In this phase, there is also delay of the melatonin rhythm, characterized by the delayed liberation and inhibition of this hormone’s secretion in more mature adolescents.^3^ Also, Andrade et al.^4^ suggested that sleep displacement (posterior waking moments) of adolescents can be attributed to the ontogenetic trend. Therefore, the addition of social, occupational and school-related morning appointments forces students to wake up early, thus contributing with sleep deprivation and daytime sleepiness.

On the other hand, sleep deprivation has substantial negative effects on motor^5^ and cognitive^6^ performance, besides humor,^7^ as well as some metabolic, hormonal and immunological variables,^8^ besides being strongly associated with daytime sleepiness.^6^ ^,^ ^9^ Excessive daytime sleepiness is characterized by the increased sensation of need for sleep, interfering in the attention and execution of routine tasks and learning situations.^10^ In a revision analysis, Pereira et al.^10^ found prevalence of excessive daytime sleepiness ranging from 7.8 to 55.8% in adolescents from different realities and countries. Therefore, excessive daytime sleepiness constitutes a discriminating variable of need for sleep.

Despite the relevance of the role of sleeping hours for the protection of daytime sleepiness, the recommendations of sleep duration per age group cannot be defined as rules, since there are major individual differences regarding the need for sleep, which compromise the generalization of these recommendations.^8^ In this sense, in a longitudinal study about the need for sleep in adolescence, Strauch and Meier^11^ identified a close connection between the group that required more sleep hours with irregular sleeping habits, shorter time in bed and more daytime sleepiness. Besides, Mercer et al.^12^ verified that these individual differences related to the need for sleep were identified before the age of 14, that is, even with relatively close sleep duration, adolescents presented with different sleep needs.

Therefore, even if the recommendations of sleep demonstrate valuable relevance in the social scope, supporting change, in several American states, during school hours, it is extremely important to verify individual differences, according to the perception of each adolescent about the need for sleeping hours, and also to observe how this variable behaves in the population of adolescents regarding their biological and perceptive specificities. So, the objective of this study was to analyze the contribution of the subjective need for sleep in daytime sleepiness in adolescents, as well as to compare matters regarding sleep, age and body mass index (BMI) among adolescents who reported sleeping enough, and those who reported needing more time of sleep.

## METHOD

This is a descriptive, observational, cross-sectional, epidemiological study. Data collection was carried out in August, 2016. The population considered for this study was 5,714 adolescents aged 14 to 19 years, regularly enrolled in high school in State schools from the city of Paranaguá, PR, Brazil. These data were granted by the State Secretariat of Education (SEED), after being approved, protocol n. 1.387.776.777. The criteria by Luiz and Magnanini^13^ were adopted for sampling calculation, with 1.96 confidence level (95% confidence interval - 95%CI), tolerable error of five percentage points, 50% prevalence (unknown outcome), and design effect of 1.5. To minimize the likely losses related to possible refusals to participate in the study, 15% were added. With these parameters, the minimum sample size for a representative sample of adolescents would include 621 students. However, the final sample was composed of 773 students aged 14 to 19 years attending two state schools in the city. The extrapolation of the minimum calculated sample was owed to the data collection by clusters of classrooms, in which all students were invited to participate in the study.

For the choice of the schools, the study and the selection of the state public schools were articulated with the Regional Center of Education (NRE) of Paranaguá. Therefore, through NRE, all schools were invited to participate in the study; however, only two agreed to. Both had more than one thousand students and included adolescents from all regions in Paranaguá.

The larger project, which this study is a part of, called “Sleep, physical activity and school performance in adolescents in the city of Paranaguá/PR”, was carried out according to the ethical principles in the Declaration of Helsinki (1964), and Resolution n. 466/2012, from the Brazilian National Health Council (2013). It was approved by the Research Ethics Committee at Universidade do Estado de Santa Catarina (CEPSH/UDESC), Report nº 1.671.544/2016. In order to participate, the students signed the informed consent form, and the parents and/or tutors signed it for adolescents aged less than 18 years.

The collection was carried out with a self-applicable questionnaire. The evaluation of daytime sleepiness was performed using the Pediatric Daytime Sleepiness Scale (PDSS), created by Drake et al.^14^ in English, with satisfactory reliability (Cronbach’s alpha=0.784),^15^ adequate reproducibility (p=0.725)^15^ and translated to Brazilian Portuguese by Felden et al.^16^ This scale is constituted of eight questions, with Likert scaled responses from zero (never) to four (always) points; the third question had reverse scoring. In this questionnaire, the higher the scoring, the higher the daytime sleepiness, with minimum of zero and maximum of 32 points. Sleep duration was represented by time in bed, which was identified considering the sleeping and waking up hours during the days with or without classes.^17^

To assess the subjective need for sleep, the following question was used: “If you could sleep longer, how long would you sleep?”, similarly to other studies.^11^ ^,^ ^12^ The responses were presented in a five point Likert scale, with the following categories: “I sleep enough, and don’t need any more sleeping hours”; “1 extra hour”; “2 extra hours”; “3 extra hours”; and “4 extra hours or more”. For purposes of analysis, the ones who answered “1 extra hour”, “2 extra hours”, “3 extra hours” and “4 extra hours or more” were classified as “Need more sleeping hours”, and those who answered “I sleep enough, and don’t need any more sleeping hours” were classified as “Sleep enough”. Besides, this question was also analyzed in an ordinal manner, ranging from zero (sleep enough) to four (four extra hours or more).

To test the validity of this question, a pilot study was carried out with students from elementary and high school at a private school of São José, SC. First, the complete questionnaire was applied to 49 adolescents aged 13 to 19 years. In one of the classes (n=16), a semantic analysis was carried out to make sure the students understood each question. Also in the pilot study, three classes were selected (n=33) to perform the analysis of reproducibility of the question using the intraclass correlation coefficient (ICC=0.702), considered acceptable by the literature.^15^ Therefore, the question was considered as reproducible and consistent.

The half-sleep phase was considered as the time that indicated half the time in bed. The formula [(sleeping time + time in bed/2) - 24] was considered to calculate the half-sleep phase. Besides, the social jetlag was used to verify the difference between the half-sleep phase on days with class and days without class.^18^ To calculate the social jetlag, the following formula was used: [half-sleep phase on days without class - half-sleep phase on days with class]. The results could be positive or negative; positive results were related to subjects who slept later on days without classes.

Also, information regarding BMI and physical activity was collected. BMI was self-reported,^19^ that is, the student reported his or her body mass and height. This variable was used continuously, and was also expressed in Z score, which corresponds to the standardized value of this index in relation to reference distribution.^20^

To analyze physical activity, we used the question extracted from the instrument called Physical Activity questionnaire for Adolescents (PAQ-A), by Kowalski et al.,^21^which was translated and validated together with the RT3 accelerometer for seven days as a reference criteria by Guedes and Guedes^22^ to Brazilian Portuguese. The question used in this study was “Which of the following situations best described your last 7 days?”, with the objective of verifying the behavior of adolescents referring to practice of physical activities in their free time. The responses of this instrument are in the five point Likert scale, in which:

1. “All or most of my free time was dedicated to activities that require little or no physical effort”.
2. “Sometimes (once or twice in the past week), the student has performed physical activities in his/her free time (for instance, practiced sports, played ball, ran, swam, danced, rode a bike, did physical exercises etc.)”.
3. “Often (3-4 times in the past week), the student has performed physical activities in his/her free time”.
4. “Pretty often (5-6 times in the past week), the student has performed physical activities in his/her free time”.
5. “Very often (7 or more times), the student has performed physical activity in his/her free time”.

The statistical analyses were carried out using the Statistical Package for the Social Sciences (SPSS), version 20.0 (IBM Corporation, Armonk, United States). After verifying that the data did not present normal distribution in the Kolmogorov-Smirnov test, we tried non-parametric analyses. Descriptive analyses of the continuous variables were carried out, represented by mean and standard deviation; categorical variables were represented by relative and absolute frequency. To compare the means between both groups, with used the Mann-Whitney U test; the chi-square test was used to analyze the association between the categorical variables.

For the correlation of the variable daytime sleepiness with the sleep variables, BMI, social jetlag, age and physical activity (continuously), we used the Spearman’s correlation test. To compare the means of the categories “sleep enough” and “need more sleeping hours”, we used the Mann-Whitney U test. With the objective of identifying the variables with higher predictive power of daytime sleepiness, regression models were calculated, using the multiple stepwise linear regression, considering the variables age, BMI (Z score), subjective need for sleep, sleeping and waking up times, time in bed, half-sleep phase during days with and without classes, and social jetlag. The assumption of normality for the residue of the regression model was met, and 95%CI was adopted for all analyses.

## RESULTS

Mean time of sleep in the sample was 7.9±1.8 hours on days with classes, and 9.2±1.8 hours on days without classes. It was observed that 74% of the adolescents claimed to need more sleeping hours. Mean score of daytime sleepiness was 14.5±5.4 points. When stratified by sex, there were no differences regarding age (p=0.218), shift (p=0.234) and time in bed on days with classes (p=0.429). However, boys presented, in average, five extra points in daytime sleepiness when compared to girls (p<0.001). It is important to mention that the mean subjective need for sleep was 1.8±1.4 hours (Table 1).

**Table 1:** Descriptive data of the sample, according to gender.

	Total (n=773)	Boys (n=388)	Girls (n=385)	p-value
Age (anos)	16.2±1.07	16.3±1.1	16.2±1.1	0.218
BMI (kg/m^2^)	21.9±3.68	22.0±3.7	21.9±3.67	0.867
BMI (Z score)	0.00±1.00	0.01±1.01	-0.01±0.10	0.867
Time to go to bed on days with classes (hours)	0:00±1:36	0:06±1:42	23:48±1:30	0.021
Time to wake up on days with classes (hours)	7:54±2:00	8:00±2:06	7:48±2:00	0.007
Time to go to bed on days without classes (hours)	1:50±2:06	2:06±2:18	1:30±1:48	0.003
Time to wake up on days without classes (hours)	11:00±1:54	11:00±2:12	10:54±1:42	0.662
Time in bed on days with classes (hours)	7:54±1:48	7:54±1:48	8:00±1:42	0.429
Time in bed on days without classes (hours)	9:12±1:48	9:00±2:00	9:24±1:42	0.001
Half-sleep phase on days with classes (hours)	3:54±1:36	4:06±1:42	3:48±1:30	0.018
Half-sleep phase on days without classes (hours)	6:24±1:48	6:36±2:00	6:12±1:30	0.053
Social jetlag (hours)	2:24±1:42	2:30±1:48	2:24±1:36	0.755
Subjective need for sleep, n (%)				
I sleep enough	208±27.0	130±33.6	78±20.3	<0.001
1 hour	131±17.0	78±20.2	53±13.8	
2 hours	190±24.6	77±19.9	113±29.4	
3 hours	96±12.5	42±10.9	54±14.1	
4 hours	146±18.9	60±15.5	86±22.4	
Subjective need for sleep, n (%)				
Sleep enough	208±27.0	130±33.6	78±20.3	<0.001
Need more sleep	563±73.0	257±66.4	306±79.7	
Daytime sleepiness (points)	14.9±5.6	21.3±12.7	16.0±5.6	<0.001
Physical activity (points)	2.2±1.2	2.4±1.2	2.1±1.1	<0.001

BMI: body mass index; values in mean±standard deviation or n (%); p-value of the chi-square test and the Mann-Whitney U test for differences between genders.

The adolescents who reported the subjective need for sleeping more woke up earlier during the week (p<0.001), later on the weekends (p=0.027), presented shorter time in bed on days with classes (p<0.001) and higher daytime sleepiness (p<0.001) when compared to adolescents who reported sleeping enough. Also, adolescents with need for sleep presented more advanced half-sleep phase during the days with classes (p=0.014), later half-sleep phase during the days without classes (p=0.044) and, consequently, more social jetlag (p<0.001) (Table 2). Besides, 62.9% of the adolescents who reported this need slept eight hours or less per night.

**Table 2:** Characteristics of the adolescents who reported sleeping enough and those who reported needing more sleep.

	Sleep enough (n=208)	Need more sleep (n=563)	p-value
Age (years)	16.22±1.13	16.24±1.06	0.705
BMI (kg/m^2^)	22.09±3.86	21.91±3.61	0.823
BMI (Z score)	0.035±1.049	-0.015±0.980	0.823
Time to go to bed on days with classes (hours)	23:59±1:50	23:58±1:32	0.601
Time to wake up on days with classes (hours)	8:22±2:00	7:44±2:01	<0.001
Time to go to bed on days without classes (hours)	1:45±2:15	1:50±2:00	0.252
Time to wake up on days without classes (hours)	10:43±1:57	11:04±1:55	0.027
Time in bed on days with classes (hours)	8:22±1:53	7:47±1:41	<0.001
Time in bed on days without classes (hours)	8:59±1:46	9:14±1:52	0.153
Daytime sleepiness (points)	11:34±4.93	16.08±5.37	<0.001
Half-sleep phase on days with classes (hours)	4:10±1:40	3:52±1:35	0.014
Half-sleep phase on days without classes (hours)	6:14±1:55	6:27±1:44	0.044
*Jetlag* social (hours)	2:03±1:43	2:35±1:39	<0.001
Physical activity (points)	2.28±1.20	2.18±1.14	0.311

BMI: body mass index; values in mean±standard deviation; p-value of the Mann-Whitney U test*.*

Among the compared variables, daytime sleepiness presented higher correlation with the subjective need for sleep (rho=0.480; p<0.001) and was not associated with time in bed, both on days with classes (rho=-0.044; p=0.225) and on days without classes (rho=0.020; p=0.589). Therefore, adolescents with higher daytime sleepiness presented more need for sleep, regardless of time in bed. Besides, positive correlations of daytime sleepiness with sleeping time (rho=0.242; p<0.001) (rho=0.221; p<0.001) and waking up time (rho=0.137; p<0.001) (rho=0.261; p<0.001) during the days with and without classes were identified, respectively. Likewise, daytime sleepiness presented positive correlation with the half-sleep phase, on days with classes (rho=0.200; p<0.001) and without classes (rho=0.266; p<0.001). The higher the physical activity during the past seven days, the lower the daytime sleepiness (rho=-0.117; p=0.001) (Table 3).

**Table 3:** Correlation of daytime sleepiness with sleep variables, body mass index, physical activity and age.

	Daytime sleepiness	
	rho	p-value
Age (years)	-0.106	0.004
BMI (Z score)	-0.053	0.153
Time to go to bed on days with classes (hours)	0.242	<0.001
Time to wake up on days with classes (hours)	0.137	<0.001
Time to go to bed on days without classes (hours)	0.221	<0.001
Time to wake up on days without classes (hours)	0.261	<0.001
Time in bed on days with classes (hours)	-0.044	0.225
Time in bed on days without classes (hours)	0.020	0.589
Subjective need for sleep (hours)	0.480	<0.001
Half-sleep phase on days with classes (hours)	0.200	<0.001
Half-sleep phase on days without classes (hours)	0.266	<0.001
Social jetlag (hours)	0.072	0.049
Physical activity (points)	-0.117	0.001

BMI: body mass index; p-value of the Spearman test.

Through the linear regression calculation, four models to explain daytime sleepiness were identified, considering the possible combinations of investigated variables, illustrated in Table 4. The first model was formed only by the subjective need for sleep (24.4%); the second, by the combination between the subjective need for sleep and the half-sleep phase on days with classes (29.3%); and the third, by the subjective need for sleep, by the half-sleep phase on days with classes and by age (30.1%). Finally, the last model was composed by the subjective need for sleep, by the half-sleep phase on days with classes, by age and by social jetlag (30.4% of the explanation). The other variables (sleep, age, BMI (Z score) and physical activity) were considered as adjustment variables to the models. It is important to mention that each extra point in the subjective need for sleep added 0.496 point in PDSS (daytime sleepiness).

**Table 4:** Linear regression analysis between daytime sleepiness and sleep variables and age.

	Daytime sleepiness (%)	Model	95%CI		Variables
	r^2^	Standardized coefficients β	Min.	Max.	p-value
Model 1					
Subjective need for sleep (points)	24.4	0.496	1.71	2.21	<0.001
Model 2					
Subjective need for sleep (points)	29.3	0.501	1.74	2.23	<0.001
Half-sleep phase with classes (hours)		0.223	0.57	1.00	<0.001
Model 3					
Subjective need for sleep (points)	30,1	0.502	1.74	2.23	<0.001
Half-sleep phase with classes (hours)		0.216	0.54	0.98	<0.001
Age (years)		-0.093	-0.82	-0.17	0.003
Model 4					
Subjective need for sleep (points)	30,4	0.489	1.68	2.18	<0.001
Half-sleep phase with classes (hours)		0.188	0.43	0.90	<0.001
Age (years)		-0.086	-0.79	-0.13	0.007
Social jetlag (hours)		0.073	0.02	0.42	0.035

95%CI: 95% confidence interval; r^2^: coeficiente of determination; adjusted analysis by the variables body mass index - BMI - (Z score), physical activity, time to go to bed (school days), time to wake up (school days), time to go to bed (days without classes), time to wake up (days without classes), time in bed (school days), time in bed (days without classes) and half-sleep phase (days without classes).

## DISCUSSION

This study verified that the perception of the adolescent on the need for sleep has contributed significantly for the explanation of daytime sleepiness. Besides, it was observed that groups with subjective need for more sleeping hours were characterized for presenting with more daytime sleepiness, waking up earlier during the week, waking up later on the weekends, and higher sleep deprivation during days with class, comparing to adolescents who considered sleeping enough. Therefore, this result provides the sleep research field with a new variable and new perspectives of diagnosis and intervention, considering the adolescents’ perception.

Regarding studies that investigated the need for sleep among adolescents, Strauch and Meier^11^ performed a study with 10 years of follow-up, including 190 adolescents initially aged between 10 and 14 years. The results indicated that adolescents needed 1.7 extra hour of sleep, which was shown in most of the sample. This result was very similar to another cross-sectional analysis (need for 2 extra hours of sleep)^12^, and with the data obtained in this study (1.8 hour). Besides, Strauch and Meier^11^ justified their results by the difficulty presented by adolescents to adapt their night time sleeping hours to the social events, and the delay in the sleep phase that commonly occurs in this stage of life, leading to higher sleep deprivation, which reflects on the need for more sleeping hours. On the other hand, Mercer et al.^12^ reported that their results go beyong, stating that the need for sleep influences on excessive daytime sleepiness and on activities of daily living, which reinforces the relationship, with high load of explanation, of the subjective need for sleep and daytime sleepiness.

Initially, the expectation was for an association between the daytime sleepiness variables and duration of sleep (represented by time in bed), as reported in other studies.^6^ ^,^ ^9^ In this analysis, however, we did not identify a correlation between these variables, but, instead, between daytime sleepiness and need for sleep. This can be explained because daytime sleepiness is not only a direct and single result of sleep duration, and it can also present other associated factors. Therefore, more robust analyses may verify the indirect relationship between these variables. In this sense, the suggestions of sleep duration for different age groups should be interpreted only as general guidelines, since ideal or sufficient sleep is intrinsic, subjective, and may change throughout life.^23^ ^,^ ^24^

Therefore, we suggest that the subjective need for sleep may have collaborated as a discriminatory variable between the adolescent who really has slept enough and the one who has not. This hypothesis was confirmed after we verified, in this study, that the subjective need for sleep presented the best predictive power of daytime sleepiness. So, as a preventive measure of daytime sleepiness and the harm caused to the adolescents’ health, the recommendation is to treat for underlying disorders, the increasing number of sleep hours (in cases when the need for more sleeping hours is reported), and adjustment of environmental factors (exaggerated use of electronic media at night, early hours of class/work), which have a negative effect on sleep.^25^

Based on the results, there was positive correlation between daytime sleepiness and the variables that constitute the sleep phase (time to go to sleep and to wake up, half-sleep phase on days with and without classes). Therefore, daytime sleepiness becomes more increased in relation to the delay in the sleep phase. This delay is common during adolescence^3^ ^,^ ^26^ ^,^ ^27^ and occurs due to hormonal changes related to maturation, which are frequent in adolescence. Therefore, the delay in the sleep phase plus the obligation to wake up early, often because of school or work in the morning, leads to daytime sleepiness. Also, despite the little explanatory power, the inclusion of social jetlag in the fourth model od daytime sleepiness explanation suggests that the incompatibility of the time of social events with the sleep needs of adolescents can constitute another path that contributes with daytime sleepiness.

Another variable that presented significant correlation with the outcome was physical activity. According to the results, the less physical activities, the higher the need for sleep among adolescents. The relationship of the short duration of sleep with physical activity has been analyzed in other studies.^9^ ^,^ ^28^ Besides, a study carried out in Santa Catarina identified that adolescents with higher prevalence of daytime sleepiness presented with higher sedentary behavior.^9^ The authors believe the adolescents with higher sleepiness end up choosing activities that require less physical effort, such as watching television and using the computer. Such behaviors are physiologically related with the suppression of melatonin production, and reduced night body temperature due to the bright lights of the media.^29^ Therefore, the findings in this study confirm, even if indirectly, the hypothesis of previous studies, which established the relationship between short sleep duration and daytime sleepiness as a result of sedentary behavior and use of electronic media.

As a positive aspect of this study, we can mention the perception of the adolescent regarding his/her need for sleep, which demonstratted 25% explanatory power of daytime sleepiness, being a valid variable, also easy to measure. It is recommended that other studies use this variable, in order to obtain more knowledge about its relationship with sleep, daytime sleepiness, sleep and health problems, besides measuring sleep and sleepiness in an objective manner.

The main limitations are the use of a “recall” questionnaire to assess the matters of sleep, which may not represent the exact fact. Likewise, the use of self-reported BMI and of only one question about physical activities may weaken the results about this variable. Also, the lack of control of sleeping problems can influence on the study’s outcome.

Finally, this study highlights the importance of the adolescents’ perception on their subjective need for sleep, inferred based on a valid and reproducible question. Besides, the subjective perception of sleep played an important role for the explanation of daytime sleepiness among adolescents, once it represents 25% of the total explanation of this variable, overcoming variables regarding sleep, age, and BMI. Besides, some characteristics of the adolescents who need to sleep more were observed, such as waking up earlier and presenting with sleep deprivation during days with classes, waking up later on weekends and reporting more daytime sleepiness, which is in contrast with those who believed to sleep enough. So, the proposal is that this question is considered in research protocols of sleep medicine and pediatrics, in order to optimize the diagnosis and the promotion of health among adolescents.
